# Guards and decoys: RIPoptosome and inflammasome pathway regulators of bacterial effector-triggered immunity

**DOI:** 10.1371/journal.ppat.1012884

**Published:** 2025-01-30

**Authors:** Haleema Sadia Malik, James B. Bliska

**Affiliations:** Department of Microbiology and Immunology, Geisel School of Medicine at Dartmouth, Hanover, New Hampshire, United States of America; Monash University, UNITED STATES OF AMERICA

## Abstract

Virulent microbes produce proteins that interact with host cell targets to promote pathogenesis. For example, virulent bacterial pathogens have proteins called effectors that are typically enzymes and are secreted into host cells. To detect and respond to the activities of effectors, diverse phyla of host organisms evolved effector-triggered immunity (ETI). In ETI, effectors are often sensed indirectly by detection of their virulence activities in host cells. ETI mechanisms can be complex and involve several classes of host proteins. Guards monitor the functional or physical integrity of another host protein, the guardee or decoy, and become activated to initiate an immune response when the guardee or decoy is modified or disrupted by an effector. A guardee typically has an intrinsic anti-pathogen function and is the intended target of an effector. A decoy structurally mimics a host protein that has intrinsic anti-pathogen activity and is unintentionally targeted by an effector. A decoy can be an individual protein, or a protein domain integrated into a guard. Here, we review the origins of ETI and focus on 5 mechanisms, in which the key steps of a pathway can include activation of a caspase by a RIPoptosome or inflammasome, formation of pores in the plasma membrane, release of cytokines and ending in cell death by pyroptosis. Survey of the 5 mechanisms, which have been shown to be host protective in mouse models of bacterial infection, reveal how distinct regulators of RIPoptosome or inflammasome pathways can act as guards or integrated decoys to trigger ETI. Common themes are highlighted and the limited mechanistic understanding of ETI bactericidal activity is discussed.

## Introduction

### Origins of effector-triggered immunity

Multicellular organisms are under constant evolutionary pressure to respond to threats by microorganisms [1]. Development of phylogenetically ancient defense strategies to counteract pathogen threat resulted in the concept of innate immunity [1]. Most of these defense mechanisms are conserved across the various phyla of life [1]. Plants rely solely on innate immune mechanisms for pathogen resistance and in this regard differ from animals who can initiate adaptive immune responses [2,3]. In 1989, Janeway shed light on innate immune mechanisms when he introduced the concept of germ-line encoded pattern recognition receptors (PRRs), which recognize conserved microbial patterns referred to as pathogen-associated molecular patterns (PAMPs), to initiate pattern triggered immunity (PTI) [4]. Plants have 2 arms of innate immune recognition, PTI being the first where membrane-associated PRRs are used to recognize bacterial components such as flagellin to initiate basal resistance, and effector triggered immunity (ETI) is the second response carried out by host defense proteins (R proteins) largely inside the cell [2,3]. If plant pathogens are successful at subverting PTI, then ETI is used for host defense [2,3]. PTI and ETI are now known to be trans-kingdom host immune strategies that are used by metazoans as well [3,5–7].

There are about 20 to 40 PRRs in mammals which respond to a diverse set of PAMPs [8]. However, pathogens are under evolutionary pressure to become specialized to evade PAMP recognition [9]. The mammalian innate immune system has developed sophisticated mechanisms to detect pathogen activity and fine tune immune responses to the level of threat and infection [6]. In this context, it’s important to distinguish between pathogenic and commensal bacteria, both of which contain PAMPs, and to distinguish pathogens that can evade PTI. Several theories have been proposed to explain this distinction. Matzinger’s “danger theory” consisted of the release of host molecules termed danger-associated molecular patterns (DAMPs), which are present the cytosol, into the extracellular milieu because of pathogen infection or sterile damage [10]. The “patterns of pathogenesis” hypothesis was proposed by Vance, Isberg, and Portnoy to further distinguish pathogenic bacteria by their location, for example, breach of cytosolic sanctity by the presence of bacterial products that would normally not be there, resulting in a strong immune response [11]. Pathogen-induced cytoplasmic alterations have also been termed “homeostasis altering molecular patterns” (HAMPs) in which innate immune sensors detect cellular imbalance as compared to microbial motifs such as DAMPs or PAMPs, allowing the host to recognize broad microbial triggers [12]. The concept of ETI also distinguishes pathogenic bacteria on the basis that they can introduce bacterial proteins called effectors in the host cytosol. Bacterial effectors have various roles in subverting host machinery for the pathogen’s benefit, but the host can recognize these activities and produce an immune response [13,14].

The basis of ETI was first introduced by Harold Henry Flor in 1942 when he proposed the “gene-for-gene resistance” hypothesis in plants [15]. Flor noticed that in the plant genus *Linum usitatissimum* (flax), some plants are susceptible while others are resistant when infected with the same microbe, *Melampsora lini* (flax rust fungus). He brought forward the concept of a dominant resistance gene (R gene) existing in the host for a corresponding pathogenic avirulence gene (Avr gene), representing a biochemical ligand-receptor–based model [15]. Resistant plants contain the R gene product which directly or indirectly recognizes the pathogenic Avr gene product, triggering a protective hypersensitive cell death response. As a result, the pathogen is unable to replicate in the plant (avirulent) [15]. Staskawicz and colleagues confirmed this hypothesis to be true when they discovered the first Avr gene (*avrA*) which belonged to *P*. *syringae* pv. *glycinea*, the causative agent of bacterial blight of soybean [16]. In the decades following this discovery, many other Avr and R gene pairs have been identified [2,3,13]. Most R proteins are cytoplasmic and belong to the class of nucleotide-binding domain leucine rich repeat proteins (NB-LRR or NLR) [2,3,13]. In this regard, the R proteins are similar to animal NOD (nucleotide oligomerization domain)/NLR proteins and evidence suggests that NLR proteins arose from a common prokaryotic ancestor [2,3,13]. Many Avr genes encode bacterial effectors which have intrinsic virulence activity and so the term ETI was coined to explain the host mechanisms associated with detecting the activity of bacterial effectors [2,13].

**Fig 1:**
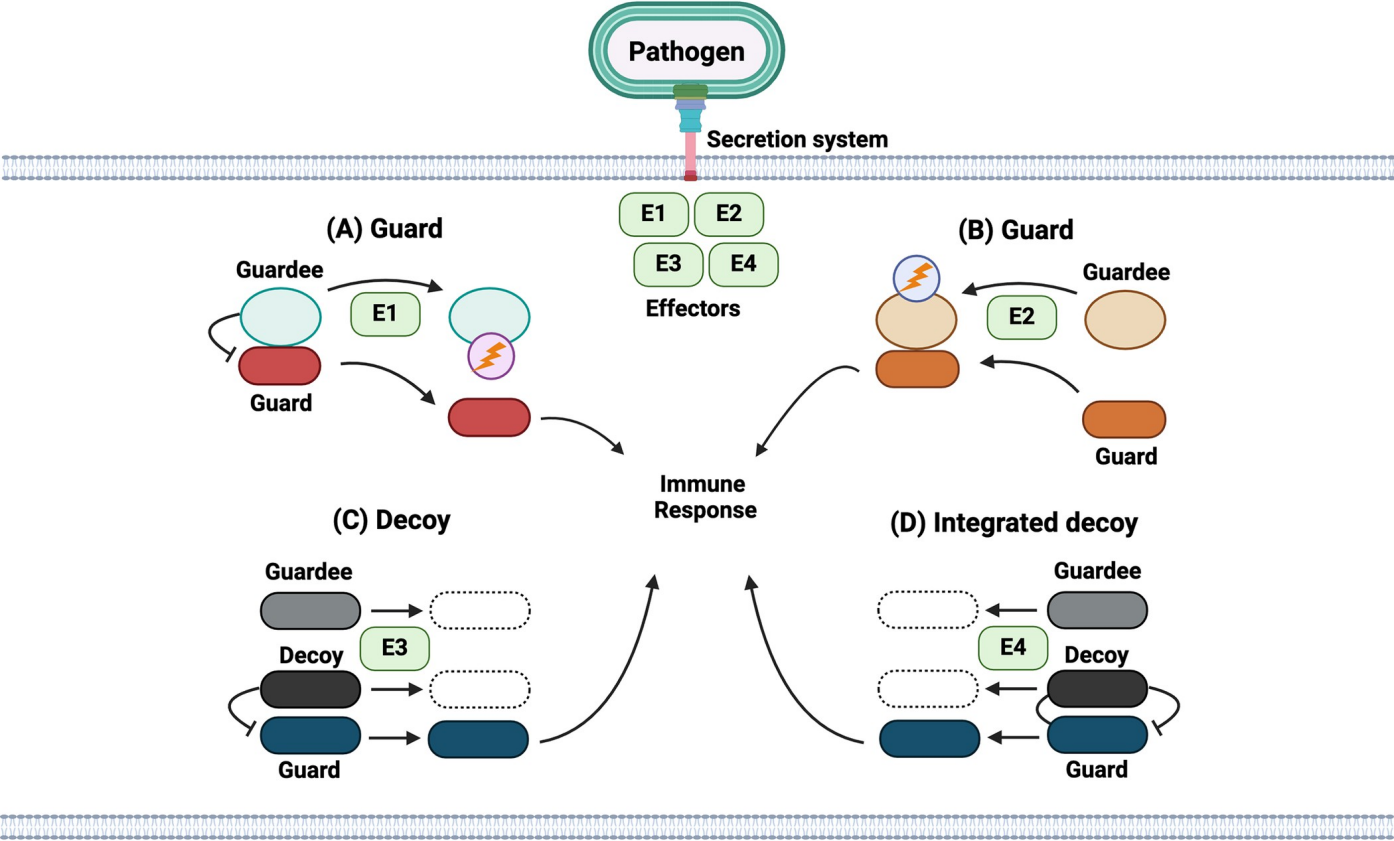
Guard or decoy mechanisms of ETI in a host cell. A generic pathogen uses a secretion system to translocate 4 effectors (E1-4) into a host cell. Effectors can be equivalent to Avr proteins and are detected by different guard or decoy proteins, which can be equivalent to R proteins. (A) The guard binds to and is inhibited by the guardee and is released to promote an immune response when E1 modifies the guardee (circle with lightning bolt). (B) Effector E2 binds to and modifies the guardee, leading to a conformational change and enhanced binding affinity for the guard protein which becomes activated. (C) The decoy mimics the effector target (guardee) and inhibits the guard. Modification of the decoy by E3 leads to its loss and activation of the guard. (D) The decoy is integrated into and inhibits the guard and modification of the decoy domain by E4 leads to its loss and activation of the guard. Figure adapted from images created with BioRender.com.

The gene-for-gene model suggested that there are direct interactions between the Avr-R proteins, but these interactions are now known to be indirect as well [2,3,13]. In 2001, Dangl and Jones expanded the concept of the gene for gene model when they proposed the “guard hypothesis” [17]. In this model, one protein “guards” the activity of another which serves as a “guardee” for the host. Upon disruption of the guardee by an effector, the guard is activated to initiate disease resistance (Fig 1) [17]. The R protein (guard) physically associates with the guardee which can be a defense component or a host protein that is modified by an effector [17]. Two variations of this model were proposed (Fig 1): (A) the R protein binds constitutively to the guardee and binding or modification of the guardee by an effector releases the R protein, which becomes activated, suggesting that the R protein is negatively regulated by the guardee; (B) an effector binds to or modifies the guardee, leading to a conformational change and enhanced binding affinity for R protein and its activation. In both predictions, there is no direct interaction between the effector and the R protein [17]. The guardee’s primary function is involved in host defense and is targeted by multiple pathogen effector molecules for successful pathogen persistence in susceptible hosts (lacking R proteins) and can be guarded by multiple R proteins as well (in resistant hosts) [17]. The guard hypothesis has been experimentally validated in plants with the discovery of many other guards and guardees [2,3].

According to the guard hypothesis, R proteins (guards) act by monitoring a protein with anti-pathogen activity (guardee) that is the target of an effector. It was proposed that some effector targets can act as decoys for effector recognition to initiate immune responses through R proteins, but these decoys don’t have intrinsic anti-pathogen activity [18]. This phenomenon serves as an extension of the guard hypothesis and was termed the “decoy model” in which the decoy mimics the effector target (Fig 1C) [18]. Decoys could arise because of gene duplication from R proteins followed by evolution or could arise independently by target mimicry [18]. The “integrated decoy model” was proposed as an extension of the decoy model in which a receptor NLR contains an integrated decoy domain (unusual non-conserved domain) that acts to bind to an effector and this receptor NLR regulates and activates a second R protein (signaling NLR) that is required to initiate resistance responses [19]. It could also be possible that a single NLR both recognizes, binds to, and initiates a response against an effector (Fig 1D), as has been shown in the case of NLRP1 as discussed below.

In summary, guard/decoy mechanisms of ETI in plants can encompass direct versus indirect means of effector recognition as discussed. Pathogen effectors can directly bind to guard proteins (integrated decoy) or effector recognition could be indirect with a guardee/decoy serving as a bridge between the effector and the guard. In the latter scenario, it can be possible that the guard does not directly interact with the guardee but is still able to sense effector disruption of it. These concepts established in plants can be applied to guard and integrated decoy mechanisms of ETI in metazoans, although the mechanistic details are different. Here, we focus on guard and integrated decoy mechanisms of bacterial ETI in mammals that have been shown to be protective in mouse infection models. Reviews that more broadly cover ETI to various pathogens in metazoans have been published [6,7,14,20,21].

### Inflammasomes and RIPoptosomes in effector-triggered immunity

ETI in mammalian host cells is commonly associated with the release of proinflammatory cytokines such as interleukin-1β (IL-1β) and a type of regulated death termed pyroptosis [22,23]. Two mechanisms of inflammatory cell death that are important in ETI, inflammasome [24,25], and RIPoptosome [26], are summarized below.

Inflammasomes mediate inflammation in response to cellular infection or stress and act as molecular platforms to assemble multiprotein complexes through protein oligomerization [24,25]. Inflammasome assembly can result in the activation of the prototypical inflammatory caspase, caspase-1 [24,25]. Once activated by an infectious or stress stimulus, pyrin domain (PYD) containing inflammasome regulators (e.g., pyrin, NLRP3) interact with the apoptosis-associated speck-like protein containing caspase recruitment domain (ASC), leading to ASC dimerization, filamentation and subsequently formation of ASC specks, which are large micrometer-sized assemblies [24,25]. ASC contains a C-terminal caspase activation and recruitment domain (CARD) through which it interacts with and recruits pro-caspase-1, leading to its activation by proximity induced autoproteolytic cleavage [24,25]. Active tetramers of p10/p20 subunits of caspase-1 process the proinflammatory cytokines IL-1β and IL-18, and the gasdermin D (GSDMD) protein [22,23]. The liberated N-terminal fragment of GSDMD forms pores in the plasma membrane to allow for the release of mature IL-1β and IL-18 [22,23]. GSDMD pores can stimulate Ninjurin 1 (NINJ1) to oligomerize in the plasma membrane, resulting in the plasma membrane rupture that is a hallmark of pyroptosis [27,28].

NLRP1 can regulate inflammasome assembly by a functional degradation mechanism [29]. Human NLRP1 contains an N-terminal pyrin domain, followed by a nucleotide-binding domain (NBD), a leucine-rich repeat (LRR) domain, a function to find domain (FIIND) and C-terminal CARD domain [30]. Mice contain 3 homologues of NLRP1 (a-c) and all lack the pyrin domain [30]. The FIIND domain undergoes constitutive self-cleavage, resulting in non-covalently linked N- and C-terminal polypeptides [20,29]. Various pathogen effectors and toxins can target and trigger degradation of the N-terminal peptide, releasing the C-terminal CARD domain containing fragment which can interact with pro-caspase-1 and promote inflammasome assembly [20,31,32].

The RIPoptosome is a multi-protein complex that promotes caspase-8 associated cell death [26] and assembles in response to perturbations in TNF receptor (TNFR1) or Toll-like receptor (TLR) signaling pathways. TNFR1 or TLR activation results in NF-κB and MAP kinase (MAPK) signaling which are essential modulators of host defense genes [33–35]. Because multiple pathogens encode effectors that disrupt TNFR1 and TLR signaling, the host has evolved a multifaceted response strategy [20,36]. Upon TNFR1 and TLR3/4 activation, complex I is assembled at the membrane which contains RIPK1 and TNF receptor associated death domain (TRADD) [37]. TRADD recruits the adapter proteins which bring in E3 ligases that ubiquitinate RIPK1. Posttranslational modifications of RIPK1 act as switches to determine the outcome of the TNFR1/TLR pathway activation. Specific polyubiquitination of RIPK1 leads to activation of TAK1 and IKKβ kinases and NF-κB and MAPK pathways [33,37]. Inhibition of TNFR1/TLR pathway signaling leads to the formation of the RIPoptosome, which consists of Fas-associated death domain (FADD) and cleaved caspase-8 [26,34,36,37], leading to caspase-8 promoted cell death via apoptosis and pyroptosis [36]. Active caspase-8 in the RIPoptosome can cleave caspases-3/7 leading to apoptosis, and GSDMD and pro-IL-1β, resulting in pore formation, IL-1β release, and pyroptosis [36]. For the latter reason, Herrmann and colleagues propose that the caspase-8 RIPoptosome should be considered an inflammasome [36] although we follow the RIPoptosome convention here.

Negative regulation of the RIPK1 caspase-8/RIPoptosome pathway is achieved by components of the TNFR1/TLR signaling pathway including direct phosphorylation of RIPK1 by IκB kinases (IKKs) and transcriptional activation of cFLIP [20,38]. Thus, the caspase-8/RIPoptosome can be activated in naïve macrophages but is blocked in cells that have experienced activation of the TNFR1/TLR signaling pathway prior to pathogen infection.

### Focus of this review and definitions

The focus of this review is on guards or integrated decoys acting as regulators of RIPoptosome or inflammasome pathways in bacterial ETI. We utilize the definition of an effector as “a microbial protein that interacts with a host cell target to promote pathogenesis” (see Box 1). Bacterial effectors are secreted proteins and typically enzymes [39–41]. ETI is defined as a protective host cell immune response to the pathogenesis activity of an effector [13,20]. Table 1 shows examples of guard or integrated decoy mechanisms in bacterial ETI in which the response has been shown to be protective in a mouse infection model and will be discussed as informative examples. PRRs such as NLRC4, AIM2, and Caspase-11/4/5 detect pathogen molecules that have gained access to the cytosol, and by activating inflammasomes, can contribute to bacterial ETI. NLRC4 associates with neuronal apoptosis inhibitor protein (NAIP) family members which bind directly to pathogen products in the cytosol such as flagellin [42,43], while absent in melanoma 2 (AIM2) binds to double-stranded pathogen DNA, to initiate inflammasome assembly [44,45]. The proinflammatory caspases-11 (murine), -4, -5 (human), directly recognize cytosolic LPS to initiate the non-canonical inflammasome pathway, leading to GSDMD cleavage [46–49]. Since these inflammasome regulators bind directly to pathogen molecules they are not covered here but have been reviewed elsewhere [6,36,42].

**Table 1:** Summary of guard or integrated decoy mechanisms of bacterial effector triggered immunity.

Guard	Guardee/ Decoy	Effector examples(s)	ETI outcome ex vivo	ETI outcome in vivo	Refs
RIPK1	TAK1, IKKβ	*Y*. *pseudotuberculosis* YopJ	Casp8 activation, pyroptosis in naïve macrophage	Reduction of bacterial CFU in organs and lethality (in RIPK1 kinase inactive mice)	[57]
Pyrin	RhoA	*Y*. *pseudotuberculosis* YopE/T	Casp1 activation, IL-1β release, pyroptosis, in LPS-primed macrophage	Reduction of bacterial CFU in organs and lethality (with Δ*yopM Yersinia*)	[77]
NLRP3	Rac2	*E*. *coli* CNF1	Casp1 activation, IL-1β release in macrophage	Reduction of bacterial CFU in blood during bacteremia	[96]
NLRP1B	NLRP1B N-terminal domain	*B*. *anthracis* LF	Casp1 activation, IL-1β release, and pyroptosis in macrophage	Reduction in lethality	[107]
GSDMA	GSDMA C-terminal domain	*S*. *pyogenes* SpeB	GSDMA activation, pyroptosis in keratinocyte	Reduction in bacterial CFU in organs and lethality	[108,109]

**Box 1. Definitions**

**Effector**: A microbial protein, usually an enzyme that interacts with a host cell target and promotes pathogenesis. Bacterial effectors are secreted proteins.

**Effector-triggered immunity**: A protective host cell immune response to the pathogenesis activity of an effector.

**Guard:** A host protein that directly or indirectly monitors the functional or physical integrity of another host protein (the guardee) and becomes activated and initiates an immune response when the guardee is modified or disrupted by the pathogenesis activity of an effector.

**Guardee:** A host protein that has an intrinsic anti-pathogen function and is targeted by an effector, leading directly or indirectly to activation of the guard.

**Decoy:** A host protein that is unintentionally targeted by an effector, leading to activation of a guard. A decoy does not have intrinsic anti-pathogen activity but instead structurally mimics a host protein that has intrinsic anti-pathogen activity.

**Integrated decoy:** A decoy that is not an individual protein but is instead a protein domain attached to a guard.

**RIPoptosome or inflammasome pathway regulator:** A protein (e.g., guard) that when activated typically forms a complex with and activates a caspase capable of cleaving gasdermin proteins leading to pyroptosis. Gasdermin proteins may act as pathway regulators that are activated by cleavage of an integrated decoy domain.

## RIPoptosome and inflammasome regulators as guards in bacterial effector-triggered immunity

### Guard RIPK1 detects inactivation of TNFR1/TLR pathway proteins by *Yersinia* YopJ

**Fig 2:**
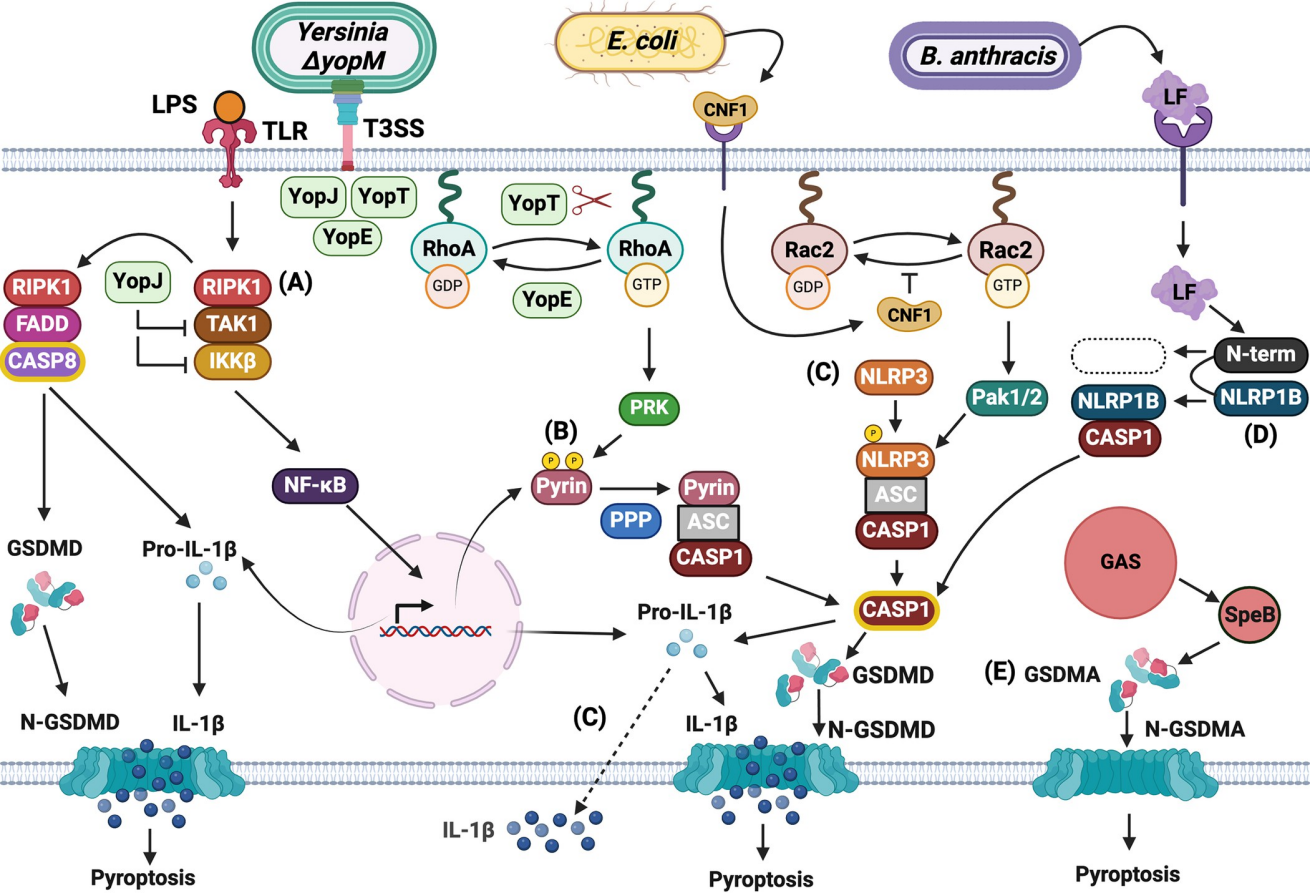
Guard or integrated decoy mechanisms of bacterial ETI in a host cell. The guard or integrated decoy in each mechanism is lettered. RIPK1 (A): Upon TLR stimulation by LPS, complex I is assembled at the plasma membrane which contains RIPK1, and leads to activation of TAK1 and IKKβ, stimulation of the NF-κB pathway and gene expression. Effector YopJ translocated by a type III secretion system (T3SS) in extracellular *Yersinia* inhibits TAK1 and IKKβ resulting in the formation of complex II (RIPoptosome) containing FADD and activated caspase-8 (Casp8) which can process GSDMD and pro-IL-1β. N-GSDMD pore formation releases IL-1β and promotes pyroptosis. Pyrin (B): RhoA inactivation by effectors YopE or YopT translocated by *Yersinia ΔyopM* removes negative regulation of pyrin by PRK and allows dephosphorylation by a phospho-protein phosphatase (PPP). Dephosphorylated pyrin forms an inflammasome with caspase-1 (Casp1) leading to release of active Casp1, N-GSDMD pore formation, processing and release of IL-1β and pyroptosis. NLRP3 (C): Extracellular *E*. *coli* secretes CNF1 toxin which is internalized by receptor-mediated endocytosis. CNF1 locks Rac2 in a GTP bound state, which activates the Pak1/2 kinases that phosphorylate NLRP3 resulting in inflammasome activation and active Casp1. Casp1 processes pro-IL-1β and IL-1β is released by a process that does not involve N-GSDMD pore formation or pyroptosis (dashed arrow). NLRP1B (D): Extracellular *B*. *anthracis* secretes LF toxin which is internalized by receptor-mediated endocytosis. LF cleaves the N-terminus of NLRP1B, and the resulting peptide is degraded by the proteosome. The liberated C-terminal CARD fragment can form an inflammasome, leading to active Casp1, N-GSDMD pore formation, processing and release of IL-1β and pyroptosis. GSDMA (E): Intracellular *S*. *pyogenes* secretes SpeB toxin which cleaves pro-GSDMA, leading to N-GSDMA pores and pyroptosis. Figure adapted from images created with BioRender.com.

RIPK1 triggers formation of the RIPoptosome when proteins in the TNFR1/TLR pathway are targeted by bacterial effectors and this ETI mechanism has been extensively studied with the pathogen *Yersinia* [36]. Pathogenic *Yersinia* species are Gram-negative, and induce a spectrum of human infections, from severe cases like plague caused by *Y*. *pestis* to mesenteric lymphadenitis inflicted by *Y*. *pseudotuberculosis* [50–52]. *Yersinia* use a plasmid encoded type III secretion system (T3SS) to inject 7 effector proteins called *Yersinia* outer proteins (Yops) into the host cytosol [50] (Fig 2). Yops disrupt crucial host cell functions, including phagocytosis and the production of proinflammatory cytokines [50]. The YopJ effector is an acetyltransferase that targets proteins in the TNFR1/TLR pathway (i.e., TAK1, IKKβ) to inhibit signaling in macrophages [53–56] (Fig 2). In naïve macrophages the caspase-8/RIPoptosome is triggered by YopJ activity against TAK1 and IKKβ as these kinases negatively regulate RIPK1 [20,36,38]. Uncontrolled RIPK1 kinase activity causes pyroptosis (Fig 2) which is essential for the host to control *Y. pseudotuberculosis* infection in a mouse infection model [57] (Table 1). Activation of the caspase-8/RIPoptosome leads to cleavage of GSDMD and GSDME and pro-IL-1β [58–62]. Intriguingly, caspase-8 activation also results in activation of caspase-1 which is independent of inflammasome components [58,63]. The mechanism of caspase-8 activating caspase-1 is not yet understood but a recent study has shown that caspase-1 activity is amplified through feedforward loops [61]. In one loop cleaved caspase-1 induces the formation of GSDMD pores which in turn activate the NLRP3 inflammasome resulting in release of IL-1β [61]. The interplay of apoptosis, which was thought to be an immunologically silent form of cell death, with pyroptosis in this case provides a sophisticated example of how the RIPK1 and caspase-8 proteins successfully guard the integrity of the TNFR1/TLR pathways. Notably, recent evidence suggests that YopJ-triggered immune responses can have mechanistic differences in murine and human macrophages infected with *Yersinia* [64], indicating that additional research is needed to understand this complex bacterial ETI.

### Guards pyrin and NLRP3 detect modification of Rho GTPases by bacterial effectors

#### Rho GTPases as guardees

The Rho GTPase family of proteins are master regulators and have important functions in mammalian cells to control a wide variety of processes such as phagocytosis, inflammation, cytoskeletal rearrangement, migration, and cell division [65,66]. Rho proteins are tethered to the plasma membrane and act as molecular switches to switch between an “on” GTP bound state to an “off” GDP bound state (Fig 2). Rho, Rac, and Cdc42 are the most well-studied subfamilies of the Rho GTPases [67]. Rho GTPase activity is regulated by GTPase activating proteins (GAPs), guanine nucleotide dissociation inhibitors (GDIs), and guanine nucleotide exchange factors (GEFs) [67]. Multiple bacterial pathogens target the Rho GTPases to subvert host innate immune pathways and hijack multiple cellular pathways [68]. Rho GTPases in such cases can act like the guardees of the host. Bacteria can secrete effectors which can either mimic GAP, GEF, or GDI proteins, or enzymatically modify Rho GTPases to either activate or inactivate them [68,69]. The innate immune system can detect such alterations to Rho GTPase activity through ETI [70,71].

#### Guard pyrin detects inactivation of RhoA by *Yersinia* YopE or YopT

Pyrin regulates the assembly of a caspase-1 inflammasome in response to inactivation of Rho GTPases by bacterial effectors and the biological importance of this interaction for ETI has been established through studies with *Yersinia* [72–74]. The *Yersinia* T3SS delivers 2 effectors, YopE and YopT, into infected phagocytes to inhibit Rho GTPases and impede phagocytosis [50–52]. YopE acts as a GAP, hastening the hydrolysis of GTP to GDP, while YopT, a cysteine protease, cleaves Rho GTPases at their C-termini, freeing them from the membrane, and preventing their interaction with downstream transducer proteins (Fig 2) [75,76]. In macrophages that have experienced TNFR1/TLR signaling prior to *Yersinia* infection the activation of the RIPoptosome by YopJ is blocked and the inflammasome regulator pyrin is produced. These regulatory steps set the stage for assembly of the pyrin inflammasome downstream of YopE- or YopT-induced Rho GTPase inactivation (Fig 2) [74,77–80]. Pyrin responds to inactivation of any one of the 3 Rho isoforms (A, B, or C) [72], and hereon we refer to RhoA for simplicity.

The pyrin inflammasome is an example of an indirect guard mechanism of ETI in mammals [72]. Pyrin responds to effector-induced inactivation of RhoA while not directly interacting with the GTPase [72]. Negative regulation of pyrin is achieved whereby GTP-bound RhoA activates 2 members of the protein kinase C superfamily, PKN1 and PKN2, which bind to and phosphorylate pyrin at S208 and S242 (S205 and S241 in mice) within a linker domain [81,82] (Fig 2). The linker is preceded by a PYD that can interact with ASC, and followed by B-box and coiled-coiled domains that allow pyrin to dimerize and form higher order oligomers like other members of the TRIM protein family [74,83]. Positive regulation of pyrin in response to RhoA inactivation is mediated by a phosphoprotein phosphatase [83]. Upon dephosphorylation pyrin is activated, interacts with ASC and forms a caspase-1 inflammasome [83] (Fig 2). Downstream of pyrin dephosphorylation, additional mechanisms are proposed to regulate inflammasome assembly, including a positive interaction step with microtubules [81,84] and binding of steroids to the C-terminal B30.2 domain in the human protein [85]. Evidence was obtained that HDAC6, an adapter protein that promotes the transport of ubiquitinated misfolded proteins by dynein motor proteins along microtubules towards the microtubule organizing centers, was important for pyrin inflammasome activation in immortalized and primary mouse macrophages [86]. However, in another study, HDAC6 was dispensable for pyrin inflammasome activation in a human monocytic cell line [87], suggesting a context-dependent role for this factor and highlighting that it remains to be determined how microtubules regulate pyrin.

A third virulence effector of *Yersinia*, YopM, plays a pivotal role in inhibiting pyrin inflammasome activation. YopM has an LRR domain structure, interacts with the PKN kinases and the RSK kinase, hijacks their activities, and maintains pyrin in its inactive state. YopM can directly bind to pyrin to mediate phosphorylation of the negative regulatory serines in the linker domain [77,78,80,88]. A *Y*. *pseudotuberculosis ΔyopM* mutant is avirulent in a mouse infection model (Table 1) [77]. The avirulence of the *Y*. *pseudotuberculosis ΔyopM* mutant is reversed in mice lacking the pyrin gene (*Mefv*^*-/-*^), highlighting the importance of the YopE/T-driven pyrin inflammasome ETI in protecting against *Yersinia* infection [77]. YopJ acetyltransferase activity has been shown to inhibit the pyrin inflammasome in macrophages and neutrophils infected with *Y*. *pseudotuberculosis* [89,90]. How YopJ inhibits the pyrin inflammasome is not known, but the block appears to be upstream of caspase-1 cleavage in macrophages and neutrophils and this activity can only be detected in the absence of YopM [89,90]. For simplicity, the interaction of YopJ with the pyrin inflammasome pathway in *Yersinia-*infected macrophages is not illustrated in Fig 2.

Several other bacterial pathogens encode RhoA-inactivating effectors which have been shown to trigger pyrin inflammasome activation either ex vivo, in vivo, or both including TcdA/B toxins of *Clostridioides difficile*, Type VI secretion system effector TecA of *Burkholderia cenocepacia*, C3 toxin from *Clostridioides botulinum*, effector VopS from *Vibrio parahaemolyticus*, *Histophilis somni* Ibpa Fic1/2, and Ptx toxin from *Bordetella pertussis* [73]. Additional experiments are needed to define if these RhoA-inactivating effectors promote bacterial ETI.

Infection with *Francisella novicida* can result in activation of the pyrin inflammasome in human monocytes [91] and murine macrophages [92]. It is unclear if and how RhoA is specifically inactivated in macrophages infected with *F*. *novicida* [92], since this pathogen is not known to secrete a Rho GTPase-inactivating effector. Aim2 is the predominant caspase-1 inflammasome activated in murine macrophages infected with *F*. *novicida* [93]. In murine macrophages infected with *F*. *novicida* ASC interacted with pyrin, Aim2, caspase-1, RIPK1, FADD, caspase-8, and other components of a complex that drives PANoptosis, a type of cell death with hallmarks of apoptosis, pyroptosis, and necroptosis [92]. In an infection model with *F*. *novicida*, mice lacking Aim2 were highly susceptible to lethal disease, while mice lacking pyrin showed intermediate mortality [92]. Taken together, these results suggest that in macrophages infected with *F*. *novicida* pyrin can be activated during PANoptosis and contribute to protection against infection in mice [92]. This appears to represent an interesting example of an inflammasome regulator being co-opted during PANoptosis and activated by a mechanism distinct from its guard and ETI function.

#### Guard NLRP3 detects activation of Rac2 by *Escherichia coli* CNF1

NLRP3 regulates the assembly of a caspase-1 inflammasome in response to multiple stimuli [94,95] including the activation of a specific Rac isoform (Rac2) by an *Escherichia coli* toxin [96]. Uropathogenic *E*. *coli* is the causative agent of urinary tract infections (UTIs) and can lead to acute renal failure [97]. Certain pathogenic *E*. *coli* can secrete the cytotoxic necrotizing factor 1 (CNF1) toxin which enters host cells through receptor mediated endocytosis (Fig 2) or outer-membrane vesicles and helps promote bacterial host cell invasion [98,99]. CNF1 deamidates Rac2 at glutamine 61, converting it into glutamic acid, and permanently locking it in a GTP bound active state (Fig 2) [98].

*E*. *coli’s* CNF1 toxin triggers protective immunity in both *Drosophila* and murine infection models, but in mice specifically this response was shown to be caspase-1–dependent [100,101]. Dufies and colleagues identified that in mice CNF1 induced activation of Rac2, leading to assembly of the NLRP3 inflammasome [96] (Fig 2). NLRP3 is regulated by subcellular localization, posttranslational modifications such as phosphorylation and ubiquitination, and association with ASC and the kinase Nek7 is critical for activation of murine NLRP3 [94,95]. In the context of CNF1 intoxication, modification of Rac2 leads to activation of the downstream kinases, p21 activated kinases (Pak1/2), which phosphorylate NLRP3 on T659 [96] (Fig 2). This phosphorylation event is important for recruitment and interaction with Nek7 and NLRP3 inflammasome activation [96]. Rac2 activation by other bacterial effectors such as the SopE GEF from *Salmonella typhimurium*, and the dermonecrotic toxin from *Bordetella*, which is a transglutaminase and blocks GTP hydrolysis of Rho proteins, were also shown to activate NLRP3 in both murine and human cells [96]. Interestingly, the Rac2-Pak-NLRP3 axis is important for clearance of Cnf1^+^ *E*. *coli* bacteraemia in mice (Table 1) and results in IL-1β secretion by macrophages by a process that is independent of GSDMD cleavage and pyroptosis (Fig 2) [96]. It could be that IL-1β interacts with membrane ruffles caused by CNF1-induced Rac2 activation but the exact mechanism of GSDMD-independent IL-1β secretion remains to be elucidated [96]. This study showed that NLRP3 guards the activity of Rac2 against activating effectors. The level of NLRP3 activation was shown to correlate with the strength of the interaction of Rac2 with Pak, representing an elegant example of how the innate immune system can modulate ETI according to the level of the threat [96].

## Inflammasome pathway regulators as integrated decoys in bacterial effector-triggered immunity

### Integrated decoy NLRP1B detects cleavage by *Bacillus anthracis* lethal factor

The concept that cleavage of an NLRP1 isoform was necessary and sufficient for caspase-1 inflammasome assembly was first established in experiments with the *Bacillus anthracis* lethal factor (LF) toxin [102,103]. *B*. *anthracis* is a gram-positive endospore-forming bacterium which causes anthrax, primarily a zoonotic disease that can also occur in humans [104]. Pathogenesis of *B*. *anthracis* depends on secretion of the anthrax toxins which consist of the LF (Fig 2) and edema factor that are transported into host cells with the help of the protective antigen [104]. Critical cellular pathways, such as the MAPK pathway, are targeted for inhibition by these toxins [104]. In mice, LF directly cleaves NLRP1B, generating an N-terminus which undergoes proteasomal degradation (Fig 2) [29,105,106]. The resulting C-terminal CARD fragment forms a caspase-1 inflammasome leading to proinflammatory cytokine production and neutrophil recruitment [29,104,105]. Mice producing the NLRP1B isoform sensitive to LF cleavage showed increased resistance to lethal infection with *B*. *anthracis* (Table 1) [107]. Interestingly, human NLRP1 does not contain the LF cleavage site [104] but is activated by enterovirus or coronavirus protease cleavage [20,31,32]. Murine NLRP1B also detects the presence of another T3SS effector, invasion plasmid antigen H 7.8 (IpaH7.8), from *Shigella flexneri* through a mechanism distinct from LF activity but dependent on proteosome degradation [29]. IpaH7.8 is an E3 ubiquitin ligase which ubiquitinates NLRP1B leading to its degradation and subsequent activation [29].

### Integrated decoy GSDMA detects cleavage by *Streptococcus pyogenes* SpeB

The discovery that cleavage of a GSDM protein by a *Streptococcus pyogenes* protease toxin results in host cell pyroptosis [108,109] indicates that immune response pathway regulators that function downstream of caspases can also serve as guards or decoys to promote ETI. *S*. *pyogenes*, or group A *Streptococcus* (GAS), is a skin pathogen which causes various acute infections ranging in severity that can be fatal as well [110]. Skin colonization and damage followed by penetration of the epithelial barrier can lead to systemic infection by GAS if not controlled earlier [110]. Secreted toxins such as streptococcal pyrogenic exotoxin B (SpeB) (Fig 2), which is a cysteine protease, are important for tissue invasion and intracellular survival [110]. The pore-forming toxin SLO allows GAS to gain access to the host cell cytosol where secreted SpeB can act on intracellular substrates [109]. Among the 5 paralogues of the pore-forming gasdermin proteins in humans (GSDMA-E), which can lead to pyroptosis when cleaved [111], GSDMA is preferentially expressed in epithelial cells. SpeB cleaves GSDMA after Q246, resulting in keratinocyte pyroptosis (Fig 2) [108,109]. Uncontrolled bacterial dissemination and cell death is observed in *Gsdma* deficient mice (Table 1), highlighting the protective role of GSDMA in controlling GAS infection in vivo [108,109]. Thus, GSDMA may contribute to ETI in response to GAS SpeB, in a manner similar to the integrated decoy model (Fig 2) [108,109]. SpeB has been shown to degrade the host proteins p62, NDP52, and NBR1 within the host cell cytosol in order for GAS to evade autophagy and replicate efficiently in the cytosol of infected cells [112]. These autophagy proteins are potential guardees of GSDMA.

## Conclusions, questions, and future directions

Using a strict definition of bacterial ETI that requires evidence for a protective response in a mouse infection model, our review of 5 mechanisms (Table 1 and Fig 2) highlights common themes in this field and focuses attention on key questions.

### Guardees or integrated decoy domains in RIPopotosome or inflammasome pathways are typically negative regulators

Guardees or integrated decoy domains for the regulators RIPK1, pyrin, and NLRP1B are all negative regulators (Fig 2). In addition, C-terminal regulatory domains of gasdermins (e.g., GSDMA) may act as integrated decoys to promote ETI (Fig 2). Remick and colleagues extend this concept by suggesting that identification of virulence factors that target negative regulators of immune responses could lead to discovery of new ETI pathways [20]. However, NLRP3 is positively regulated by the guardee Rac2 (Fig 2) [96] indicating there can be exceptions to this common theme.

### Bacterial ETI can drive an evolutionary arms race between host and pathogen

Two inflammasome-dependent ETI mechanisms covered in this review provide notable examples of evolutionary arms races. In the case of the YopE/T-driven pyrin inflammasome ETI (Fig 2), *Yersinia* likely evolved YopM to maintain virulence. Population genetics suggest that historic plague pandemics in turn selected for gain of function mutations in the pyrin gene (*MEFV*) in carriers in the human population to overcome the YopM block of pyrin inflammasome activation [88]. This heterozygous advantage in carriers came with a disadvantage as homozygous gain of function mutations in *MEFV* are responsible for one of the most common autoinflammatory diseases, Familial Mediterranean Fever [113,114].

A second example of an inflammasome-dependent ETI arms race comes from NLRP1. The N-terminal decoy domain of NLRP1B that is recognized by *B*. *anthracis* LF (Fig 2) and triggers protection against anthrax is not present in humans. The decoy domain in human NLRP1 appears to be evolving under positive selection to acquire other targeting motifs, such as those recognized by viral proteases [20,31,32]. Human NLRP1 contains an N-terminal PYD that is dispensable for inflammasome formation due to the presence of the C-terminal CARD. It has been hypothesized that this domain is targeted by a PYD-targeting effector that has yet to be identified [20].

### Bacterial ETI pathways can be differentially regulated in space and time

Naïve macrophages can detect the *Yersinia* effector YopJ and trigger the caspase-8 RIPoptosome because RIPK1 is not inhibited from prior TNFR1/TLR signaling in these cells. Conversely, naïve macrophages are unable to detect *Yersinia* effectors YopE/T because TNFR1/TLR signaling is required for production of pyrin. On the other hand, in macrophages that have been primed by TNFR1/TLR signaling prior to *Yersinia* infection, RIPK1 is inhibited, and pyrin is produced, and thus the translocated effectors YopE/T selectively trigger the pyrin inflammasome ETI (Fig 2). Protective ETI dependent on RIPK1 or pyrin have been detected in mice infected with *Yersinia* (Table 1), indicating that both responses can benefit the host in vivo. Interestingly, the ETI against *Y*. *pseudotuberculosis* dependent on RIPK1 kinase activity is detected in oral but not systemic mouse infection [57]. The ETI against *Y*. *pseudotuberculosis* Δ*yopM* dependent on pyrin is detected in a systemic mouse infection model [77] but has not been tested in oral challenge. It is possible that RIPK1 kinase-dependent ETI is triggered in naïve phagocytes that *Y*. *pseudotuberculosis* encounters and injects YopJ into at early stages of infection in intestinal or mesenteric lymph node tissues [57]. At later stages of infection when *Y*. *pseudotuberculosis* has disseminated to organs and is forming pyogranulomas in tissues, the pyrin inflammasome may be preferentially triggered in primed phagocytes injected with YopE/T. Thus, distinct ETI pathways to the same pathogen may be differentially regulated in a location and temporal manner during host infection.

### Effector-triggered immune responses can promote pathogenesis

This review has focused on effector-triggered immune responses that are host protective but there are also examples with the opposite outcome. Pathogens can stimulate inflammation to outcompete commensal microbes or to promote their own transmission [115]. In some cases, these immune responses can lead to uncontrolled inflammation and damage. *Pseudomonas aeruginosa* infections can cause severe pulmonary damage [116]. Inflammation during *P*. *aeruginosa* infection in a mouse model was shown to be IL-1β driven [117]. Sun and colleagues found that the *P*. *aeruginosa* type 2 secretion system effector metalloprotease, LasB, cleaves pro-IL-1β on a site distinct from caspase-1, thus removing the inhibitory N-terminal domain. Production of mature IL-1β leads to neutrophilic inflammation and lung damage [117]. LasB is abundantly present in the lung microenvironment during *P*. *aeruginosa* infection [117]. The inhibitory N-terminal domain of pro-IL-1β can be considered an integrated decoy that is targeted by the effector LasB, and in this case the outcome is pathogenic.

Another example of an effector-triggered immune responses that is pathogenic involves the detection of viral protease activity by CARD8. CARD8, like NLRP1, can be activated by an integrated decoy mechanism that releases a C-terminal CARD domain upon viral protease cleavage [118–121]. In a humanized mouse model of infection, HIV-1 protease cleavage of CARD8 in CD4^+^ T-cells results in caspase-1 inflammasome assembly and pyroptosis, leading to depletion of these cells, which is one of the hallmarks of disease progression in AIDS [122]. This result also highlights the importance of cell type in determining the outcome of effector-triggered immune responses in causing pathogenesis versus ETI, as protease cleavage of CARD8 in the endothelium has been implicated in anti-viral immunity [119].

### What are the protective mechanisms of bacterial ETI?

The 5 protective ETI mechanisms reviewed here (Table 1) have release of IL-1β (and probably IL-18) and/or pyroptosis in common (Fig 2). Release of IL-1β and IL-18 could contribute to host protection against primarily extracellular pathogens such as *Y*. *pseudotuberculosis*, *E*. *coli*, and *B*. *anthracis* (Fig 2). These cytokines help to recruit and activate bactericidal phagocytes to control infection. Pyroptosis resulting from GSDM pores can result in release of other proinflammatory proteins such as IL-1α [123]. Pyroptosis can remove an intracellular niche and trap bacteria inside macrophages, which might make *S*. *pyogenes* (Fig 2) susceptible to efferocytosis and killing by neutrophils [124]. Recent studies show that RIPK1- or pyrin-dependent pyroptosis can occur in murine neutrophils infected ex vivo with *Y*. *pseudotuberculosis* [62,90]. Activation of the caspase-8 RIPoptosome in neutrophils resulted in cleavage of GSDME, release of IL-1β and pyroptosis [62]. Neutrophil release of IL-1β was implicated in RIPK1-dependent ETI in a mouse infection model [62]. Infection of primed murine neutrophils with *Y*. *pseudotuberculosis* Δ*yopM* resulted in activation of the pyrin inflammasome, GSDMD pore formation, IL-1β release and pyroptosis [90]. In addition, extrusion of neutrophil extracellular traps (NETs) downstream of pyroptosis was detected in primed murine neutrophils infected with *Y*. *pseudotuberculosis* Δ*yopM* [90]. NETs can trap and kill extracellular bacteria [125] and use of mice deficient in NET extrusion yielded data suggesting a protective role against *Y*. *pseudotuberculosis* Δ*yopM* [90]. Thus, it is possible that neutrophils play an important role in bacterial ETI.

### Future directions

For the bacterial ETI field to move forward it is important to identify new effector-triggered immune responses that are protective, but this can be challenging. For example, host-adapted bacterial pathogens can encode inhibitors of effector-triggered immune responses that need to be inactivated before the ETI can be discovered and studied. Development of genetic screens that can uncover bacterial inhibitors of ETI could be useful in overcoming this barrier. There can be mechanistic differences in regulation and outcomes of effector-triggered immune response pathways between murine and human cells and more work is needed to understand the biological impact of these distinctions. Comparison of mice with humanized immune systems to conventional controls in infection models will help define how bacterial ETI mechanisms may differ between host cell types in vivo. Future studies are needed to gain deeper insights into bactericidal mechanisms of ETI. Important goals are to determine which cell types and immune responses are bactericidal, and to define if there is relevant cross talk between different cell subsets in bacterial ETI. Results of these studies may also inform the development of novel countermeasures against pathogens. In this context, host-directed therapeutics that enhance ETI could be useful for treating ongoing infections. Small molecules that selectively activate guards or decoys could be helpful in this case. Alternatively, attenuating pathogens by removing ETI inhibitors might be a good strategy to develop live vaccines that generate robust immunity.
